# The Cortico-Basal Ganglia-Cerebellar Network: Past, Present and Future Perspectives

**DOI:** 10.3389/fnsys.2019.00061

**Published:** 2019-10-30

**Authors:** Demetrio Milardi, Angelo Quartarone, Alessia Bramanti, Giuseppe Anastasi, Salvatore Bertino, Gianpaolo Antonio Basile, Piero Buonasera, Giorgia Pilone, Giuseppe Celeste, Giuseppina Rizzo, Daniele Bruschetta, Alberto Cacciola

**Affiliations:** ^1^Department of Biomedical, Dental Sciences and Morphological and Functional Images, University of Messina, Messina, Italy; ^2^IRCCS Centro Neurolesi “Bonino Pulejo”, Messina, Italy; ^3^I.S.A.S.I.E. Caianello, National Research Council, Messina, Italy

**Keywords:** cerebellum, connectomics, globus pallidus, substantia nigra, tractography

## Abstract

Much of our present understanding of the function and operation of the basal ganglia rests on models of anatomical connectivity derived from tract-tracing approaches in rodents and primates. However, the last years have been characterized by promising step forwards in the *in vivo* investigation and comprehension of brain connectivity in humans. The aim of this review is to revise the current knowledge on basal ganglia circuits, highlighting similarities and differences across species, in order to widen the current perspective on the intricate model of the basal ganglia system. This will allow us to explore the implications of additional direct pathways running from cortex to basal ganglia and between basal ganglia and cerebellum recently described in animals and humans.

## Introduction

The brain is a complex network consisting of a huge number of neurons (~10^11^) segregated in spatial regions with similar cytoarchitecture and functional features. Identifying anatomical physical pathways between the various structures of the brain has always been a major challenge in neuroscience. Neuronal connectivity patterns can be investigated at different levels of scale: (i) the microscale allows to study single synaptic connections linking two or more individual neuronal cells, providing a detailed anatomical description of the basic substrates of the cerebral microcircuits; (ii) at the mesoscale level, where brain connectivity is investigated at the level of columns and mini-columns; and (iii) at macroscale level that explore large-scale anatomical connectivity patterns focusing on the inter-regional white matter pathways connecting distinct neuronal populations (Sporns, 2011).

The current knowledge about the short-, medium- and long-range neuroanatomical connections of the basal ganglia system comes from both invasive and non-invasive experimental techniques applied respectively in animals and humans.

The basal ganglia are a group of subcortical nuclei which integrate information from widespread cortical areas and in turn project their outputs back to the cerebral cortex (Alexander et al., 1990). Considering their pivotal role in motor and non-motor functions, the basal ganglia have been a main topic of interest in the field of basic and clinical neurosciences. Basal ganglia connections have been widely studied and different models of basal ganglia circuitry have undergone major revisions during the last decades (Nelson and Kreitzer, 2014).

The present review aims at providing a comprehensive overview on the interactions between the cerebral cortex, the basal ganglia and the cerebellum in order to better understand how such interplay contributes to specific attributes of motor and non-motor behavior and to the pathophysiology of basal ganglia disorders.

We will first discuss the most common invasive and non-invasive techniques to study brain connectivity respectively in animals and humans. We will then review the traditional models of basal ganglia anatomy and circuitry highlighting similarities and differences across species. Finally, we will widen the current perspective on basal ganglia connectomics providing a new challenging, comprehensive and integrated cortico-basal ganglia-cerebellum model.

## History of Basal Ganglia Concept

The presence of structures at the basis of the human brain had already raised the attention of many scientist from the antiquity to the 19th century; early anatomical depictions of the basal ganglia appear in the works of classical anatomists such as Galenus or Vesalius; the use of the term “corpus striatum,” to refer to the large subcortical masses located nearby the cerebral ventricles, is attested early in Thomas Willis *“Cerebri Anatome”* (1664) (Parent, 2017). Most of the actual nomenclature used to describe basal ganglia structures comes from authors of late 18th and early 19th century; in particular, terms such as “globus pallidus,” “external capsule,” “internal capsule,” “lenticular nucleus” are introduced in the classical treatise of Karl Friedrich Burdach (Parent, 2013). In the same period, structures such as the substantia nigra and the subthalamic nucleus (Luys, 1868) were described. The term *Basal Ganglia* has been originally proposed by Sir David Ferrier in a highly challenging and comprehensive masterpiece of the 19th century on the yet unraveled brain structure and function, “The functions of the brain” (1887). In this treatise, Ferrier writes that “the basal ganglia—the *corpora striata* and *optic thalami*—are ganglionic masses, intercalated in the course of the projection system of fibers which connect the cortex with the *crura cerebri*, and through these with the periphery. The corpora striata are the “ganglia of interruption” of the projection system of the foot or basis of the crus, an anatomical indication of their motor signification” (Ferrier, 1887).

After that, a wide corpus of research has been focused on basal ganglia structure and function both in health and in disease. The last half of the 20th century has seen the rise of neuroanatomical tracing techniques, that allowed for a complete description of basal ganglia anatomy and connectivity in different animal species. In the last 20 years, these techniques have been paralleled by neuroimaging techniques focused at reconstructing white matter anatomy of the human brain. An overview of strengths and limitations of such techniques will be provided below.

## Invasive and Non-invasive Approaches to Study Anatomical Connectivity

### Traditional Anterograde and Retrograde Tract Tracing

Despite several efforts have been made to study brain and basal ganglia functional anatomy, the most recent breakthroughs occurred with the development of various powerful neuronographic methods, introduced in late 20th century, which have allowed to describe the close interrelation between the core structures of the basal ganglia and to set the ground basements of the current ideas on the basal ganglia circuits.

Degeneration and tract-tracing approaches are among the most common methods applied in animal studies. Highly localized lesions, leading to Wallerian degeneration, combined with stains that selectively color degenerating neuronal cell bodies and axons have been helpful in the past to trace neural pathways (Johnson, 1961; Afifi et al., 1974). However, degeneration techniques are limited by the low accuracy to determine the exact location of axonal terminals and by the fact that not all the neurons show marked degeneration after a lesion. Taking into account such limitations, the second half of the 20th century has been characterized by a methodological innovation based on the axonal transport of tracers. Anterograde tract-tracing allows to identify the axons terminations by injecting chemical tracers and dyes which are incorporated into macromolecules by the neuronal cell bodies and then carried to the end of the axons. Another widely used tract-tracing strategy is retrograde tracing: a molecular marker (i.e., horseradish peroxidase enzyme) injected into the area of axonal terminations is carried *via* the retrograde axonal transport towards the cell body thus revealing the origin of the neuronal pathway (Köbbert et al., 2000; Raju and Smith, 2006; Schofield, 2008). Regardless of the transport direction, time must be considered to allow the tracer reaching its destination and then to proceed with tracer detection using fluorescent light or immunohistochemistry. Although the astonishing findings revealed by experimental tract-tracing in animals, this technique did not have successful application in the post-mortem human brain due to slow rate of diffusion (Beach and McGeer, 1987; Haber, 1988). In addition, both anterograde and retrograde tract-tracing are prone to limitations, considering different potential sources of false-positive and false-negative results. As a matter of fact, it is possible that tracer injections may spread beyond the target or involve adjacent pathways; also, it is possible that retrograde tracers are uptaken by fibers of passage, producing false-positive results (Reiner et al., 2000; Van Haeften and Wouterlood, 2000). Furthermore, when using biotinylated dextran amine (BDA) for anterograde tracing care should be taken due to the possible retrograde trafficking and the subsequent anterograde transport into neuronal collaterals (Reiner et al., 2000).

On the other hand, false-negative findings may derive considering the inability to label all neurons in a population in any given study. Another potential source of false-negative findings is that it might not be possible to identify the colocalization of markers especially when the neuronal structures are tiny, due to either imperfect antibody penetration or disproportional concentration of antigens (Reiner et al., 2000; Van Haeften and Wouterlood, 2000). Despite the outstanding historical importance of tract-tracing and its actual advantages, these limitations led to the development of new, more precise tracing methods.

### Neuronal Tracing by Neurotropic Viruses

Beyond conventional tracers, neurotropic viruses have the great potential to exploit the connectivity of neural circuits; viral replication amplifies the signal at each step of the process; moreover, viral tracers are able to traverse multisynaptic pathways. These features allow a more precise individuation of anatomical connections and to distinguish between direct and indirect projections. Albeit several neurotropic viruses exist, only two major classes, the herpes and rabies viruses, have been traditionally employed to experimentally track neuronal pathways. While such classes of viruses are substantially different, they do share an envelope structure and the ability to infect neurons and to spread along the nervous system. Ugolini et al. (1987) demonstrated for the first time ever that the herpes simplex virus type 1 (HSV 1) could be used to trace neural connections across at least two synapses in rodents, thus paving the way for further development of virus tracing in non-human primates (Hoover and Strick, 1993; Middleton and Strick, 1994). As major limitations, HSV 1 induces rapid neuronal degeneration and may spuriously spread to glial and other neuronal cells. As a consequence, attempts to limit the local spread do not allow to trace further than second-order neurons (Kaplitt and Loewy, 1995). By contrast, rabies viruses do not induce neuronal degeneration and are able to detect neuronal connections across an unlimited number of synapses (Ugolini, 2011). However, major drawbacks in using viruses to label multisynaptic connections are the low speed of the viral transport, paralleled by their fast-lethal effects on the experimental animal, that dies for the infection after a short time. Consequently, and considering that at least 2 days are needed to label first-order neurons, higher-order neurons are labeled only after 12 h or more from that time (Aston-Jones and Card, 2000). Therefore, tracking a neuronal network consisting of, e.g., seven synapses, could take approximately up to 1 week.

However, despite all the above-mentioned limitations virus transneuronal tracing still remains the gold standard approach to map axonal connections in animals. On the other hand, the application of such invasive tracking methods is elusive when applied to the human brain.

### Non-invasive Neuroimaging Approaches for the Human Brain

The great success of neuroanatomical tracing has boosted the research on neuronal connectivity based on animal models. However, translating such findings from animals to the human brain posits some non-negligible theoretical issues: it forces the assumption that brain structures of interest are relatively conserved in the human brain, and it does not account for inter-specific differences. During the last decades, the progress of magnetic resonance imaging (MRI) has allowed the development of neuroimaging approaches as an alternative modality to assess morphological neuronal connectivity patterns in living humans. Diffusion-weighted magnetic resonance imaging (DWI) and tractography have been successfully employed to model and infer white matter bundles’ trajectory of white matter bundles together with their microstructural properties (Milardi et al., 2016b, 2017; Cacciola et al., 2017a,c; Calamuneri et al., 2018; Rizzo et al., 2018; Arrigo et al., 2019). Despite these techniques have lower spatial resolution than chemical and virus tract-tracing and they are not able to estimate the directionality of neural pathways, they do provide the only chance to explore anatomical connectivity *in vivo* and non-invasively in the human brain (Chung et al., 2011).

DWI allows to measure water molecules diffusion along different directions. Considering the impermeable nature of axons, water diffusivity is highly directional (anisotropic) being constrained to the main axonal direction; therefore DWI indirectly evaluates white matter microstructure (Basser et al., 1994, 2000). Assuming that such local diffusion is explained by a three-dimensional Gaussian process, the main axis of the diffusion ellipsoid corresponds to principal diffusion direction and its fractional anisotropy corresponds to the degree to which diffusion is preferred along this direction over other directions. Therefore, by computing the principal local diffusion direction within the single voxels and attempting to infer specific spatial axonal trajectories, tractography can be used to map and reconstruct main fiber bundles at a system level (Alexander et al., 2007). Classical diffusion-weighted images used for tractographic reconstruction usually have a voxel resolution of 2 × 2 × 2 mm^3^ which is notably higher than the axonal diameter (Jbabdi and Johansen-Berg, 2011), whilst traditional anatomical tracers can track the projections of single axons. Another major drawback of tractography is the inability to determine the polarity of a given connection and thus to establish whether a given fiber pathway is afferent or efferent (Parker et al., 2013).

In addition, simple diffusion signal modeling approaches cannot reliably disentangle the complex white matter architecture consisting of twisting, bending, crossing and kissing fibers thus failing in representing any of their orientations. To overcome this issue, “model-free” approaches have been developed in the last decade, such as Diffusion Spectrum Imaging (DSI; Wedeen et al., 2005), Q-ball Imaging (Tuch et al., 2003) and Constrained Spherical Deconvolution (Tournier et al., 2007).

Despite the above-mentioned limitations, DWI and tractography are the only existing techniques able to investigate anatomical connectivity in the human brain *in vivo* and non-invasively. Indeed diffusion tractography has been extensively recognized as the first “*in vivo* dissection” approach to map the major fiber bundles in the human brain with extreme precision as well as to show the existence of new associative pathways that have been subsequently replicated using the traditional post-mortem Klingler dissection (Klingler, 1935; Klingler and Gloor, 1960). For instance, the increasing use of tractography has boosted our understanding of the morphological shape of the major long-range white matter pathways (Catani et al., 2005; Parker et al., 2005; Yagmurlu et al., 2016), consequently confirmed by post-mortem dissection in the human brain (Lawes et al., 2008; Yagmurlu et al., 2016). Last but not least, tractography has allowed to develop several atlas of the human brain (Mori and van Zijl, 2007; Oishi, 2011; Catani and Thiebaut de Schotten, 2012). As a final remark, the anatomical validity and reproducibility of DWI tractography have been assessed *in vitro* in a highly gyrated model of the porcin brain, demonstrating that tractography is able to reliably detect specific white matter pathways and therefore to be a powerful tool in investigating anatomical brain connectivity (Dyrby et al., 2007).

## Inter-Species Commonalities and Differences in the Basal Ganglia Network

Despite the basic basal ganglia anatomy and connectivity are well preserved across most species, from rodents to non-human and human primates (Reiner et al., 1998; Stephenson-Jones et al., 2012), some meaningful interspecific topographical and functional variations need to be carefully addressed. The basal ganglia have been observed in all amniote species; the basic organization of these telencephalic nuclei seems to be phylogenetically conserved, since evidences of a remarkable similarity between lampreys, the oldest now-living vertebrates, and mammals have been carefully described (Grillner and Robertson, 2016). This supported the view according to which rudimentary basal ganglia were already present in the vertebrate’s common ancestor.

In mammals, the basal ganglia demonstrate a much more extensive interaction with the cerebral cortex (Reiner et al., 1998). Furthermore, the basal ganglia seem to maintain the same circuit organization, namely the presence of input nuclei, output nuclei and modulatory stations through which information is funneled and processed (Gerfen et al., 1987a,b; Smith and Parent, 1988; Alexander et al., 1990). Indeed, several neuroanatomical and neurophysiological insights gained studying rodents have been lately confirmed in primates. However, some remarkable differences both in the macroscopic and microscopic anatomy need to be addressed.

From the gross anatomy perspective, the striatum could be divided into a dorsal and a ventral compartment; these two divisions lack of a clear boundary but greatly differ in their connectivity profiles (Haber and Knutson, 2010). In rodents, the dorsal striatum is named neostriatum and it could be divided into a dorsomedial and a dorsolateral part, whilst in monkeys and humans it is divided into caudate nucleus and putamen (Grillner and Robertson, 2016). Structural separation of the striatum into caudate nucleus and putamen by the internal capsule in primates does provide a clear functional distinct segregation of the cortical inputs to these two main structures. Although the caudate nucleus is traditionally associated with cognitive functions and the putamen with motor functions, both structures receive widespread afferents from the cerebral cortex, see Haber (2016) for an extensive review. Rodents, instead, lack of such structural separation within the dorsal striatum.

In parallel, the same considerations could be made for the difference in the Globus Pallidus (GP) gross anatomy between rodents and primates. Both in human and non-human primates, the internal (GPi) and external (GPe) segments of the GP are structurally divided by the internal lamina and are placed close to each other. On the other hand, in rodents, the GPi functional homologous, termed entopeduncular nucleus, is mostly embedded in the internal capsule (Carter and Fibiger, 1978) whilst the GPe homolog is termed simply as GP.

Moreover, a striking difference between primates and rodents is represented by the cerebral cortex, which constitutes one of the main interacting systems with the basal ganglia. In primates, the need for more complex motor tasks and sensory integration has allowed the development of large and architecturally complex association areas (Preuss and Goldman-Rakic, 1989, 1991). According to recent works, this difference would make the old world monkeys the most valuable animal model to study function and disease of the basal ganglia (Smith and Galvan, 2018).

A main difference is that in rodents, cortico-spinal pyramidal neurons directly synapse on the striatum; in primates, on the other hand, cortico-spinal and cortico-striatal descending systems are totally segregated (Parent and Parent, 2006; Kita and Kita, 2012; Smith et al., 2014).

Moreover, differences in volume, distribution and number of neurons of the basal ganglia among mammals have been described, underlining substantial differences between rats and non-human primates and subtle variations between humans and monkeys (Hardman et al., 2002).

Despite such morphological differences, the organization of the main afferent and efferent systems of the basal ganglia network is almost similar across species. With some limitations, and paying attention to inter-species differences, rodents still constitute a valuable model to study basal ganglia in physiology and disease (Hooks et al., 2018; Miyamoto et al., 2019).

## Traditional Circuits of the Basal Ganglia Network

**Figure 1:**
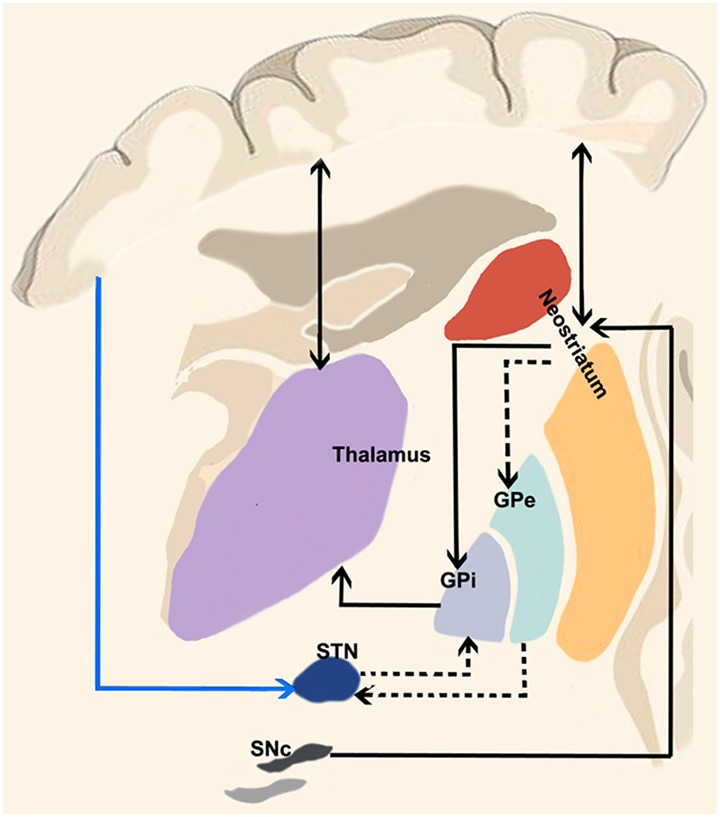
“Classical” cortico-basal ganglia-cerebellar pathways. The most basic circuit model of basal ganglia function involving the “direct” and “indirect” pathways originally proposed by Albin et al. (1989). Red lines highlight the “direct” pathway funneling information from the cerebral cortex to the striatum and then to internal segment of the globus pallidus/pars reticulata of the substantia nigra (GPi/SNr) *via* GABAergic inhibitory projections thus selectively reducing GPi/SNr activity and releasing the thalamocortical circuits involved in motor pattern generators. The dotted black lines depict the “indirect” pathway: when excited by the glutamatergic inputs of the cerebral cortex, striatal medium spiny neurons (expressing D2 receptors) allow the cells of the striatal matrix to send inhibitory signals to the GPe, thus exerting its tonic GABAergic inhibition on the subthalamic nucleus (STN). Therefore, the glutamatergic neurons of the STN can excite the GPi/SNr thus suppressing thalamic activity on the cerebral cortex and increasing inhibitory influences on the upper motor neurons. More recently, a “hyperdirect” pathway has been described (blue line between the cerebral cortex and STN), conveying excitatory stimuli from motor, associative and limbic brain areas on the STN, bypassing the “indirect” inhibitor circuit and leading to excited GPi/SNr activity.

The most basic circuit model of basal ganglia function involving the “direct” and “indirect” pathways has been originally proposed by Albin et al. (1989) and it has represented the cornerstone of our knowledge on basal ganglia function for two decades (Figure 1). More recently, DeLong and Wichmann (2007) have suggested that the output nuclei—the GPi and the SNr—exert a tonic firing to the intralaminar and ventral motor nuclei of the thalamus which in turn regulate motor-related areas in the cerebral cortex (DeLong and Wichmann, 2007) influencing desired and unwanted behaviors.

A third fundamental pathway, the so-called “hyperdirect pathway” of the basal ganglia circuitry has been recently identified. Although the subthalamic nucleus (STN) has been considered for many decades one of the relevant nodes of the “indirect” pathway, it also receives direct signals from the cerebral cortex (Nambu et al., 2000).

When a given motor pattern is computed by cortical motor areas, it is first conveyed to the basal ganglia *via* glutamatergic projections with the purpose of releasing the intended movement and suppressing the unintended ones. The “direct” pathway funnels information from the striatum to internal segment of the globus pallidus/pars reticulata of the substantia nigra (GPi/SNr) *via* GABAergic inhibitory projections thus selectively reducing GPi/SNr activity and releasing firing from thalamocortical neurons.

Along with the initial signal to the striatum, the cerebral cortex suppresses surrounding or competing motor patterns. This activity is known to be mediated by the “indirect” and “hyperdirect” pathways. When excited by the glutamatergic inputs of the cerebral cortex, striatal D2 receptors allow the cells of the striatal matrix to send inhibitory signals to the GPe which normally exerts a tonic GABAergic inhibition on the STN. Therefore, the glutamatergic neurons of the STN can then excite the GPi/SNr thus suppressing thalamic activity on the cerebral cortex and increasing inhibitory influences on the upper motor neurons (DeLong and Wichmann, 2007, 2009; Stinear et al., 2009; Noorani and Carpenter, 2014). Moreover, the glutamatergic “hyperdirect” pathway, conveying excitatory stimuli from motor, associative and limbic brain areas on the STN (Nambu et al., 2002) triggers GPi/SNr activity (Figure 1) bypassing the indirect pathway. This latter view is supported by the fact that cortical neurons projecting to GPe appear to be in a different group than those projecting to STN (Kita and Kita, 2012). The following inhibition of the thalamocortical projections suggests therefore a major role of the hyperdirect pathway in holding back movements (Wessel et al., 2016).

## Neurophysiological Insights on Basal Ganglia Function

A possible electrophysiological correlate of basal ganglia activity in the human brain is the Bereitschaft potential, also known as readiness potential (RP), a slow negative electroencephalographic (EEG) activity that usually precedes self-paced movements (Shibasaki and Hallett, 2006). The RP has been initially considered as an electrical phenomenon originating from cortical activity which occurs before both simple and complex motor tasks (Rektor et al., 1994, 1998, 2001a). However different evidences suggest that RP may be recorded also from subcortical structures such as striatum and thalamic nuclei (ventral intermediate nucleus VIM, ventroposterior nucleus VP; Rektor et al., 2001c). In particular, latencies of RP recorded in the putamen precedes those recorded by electrodes implanted in cortical motor areas (Rektor et al., 2001a). Following investigations conducted on patients implanted in caudate nucleus, putamen and GPi demonstrated that these regions are potential substrates for RP generation (Rektor et al., 2001b); this is in line with previous evidences of disrupted RPs after lesions in the basal ganglia (Dick et al., 1989) and suggests that cortico-basal ganglia-thalamo-cortical reverberating circuits may be involved in the generation of RP. Moreover, a P3-like activity has been recorded in basal ganglia and in cortical motor and premotor areas during a multimodal evoked related potential (ERP) stimulation paradigm aimed at investigating electrical activity related to cognitive processing of sensorial stimuli (Rektor et al., 2003). This suggests a possible interplay of cortical areas and basal ganglia during cognitive processing.

## Beyond the Direct, Indirect and Hyperdirect Pathways

One of the main aims of the present review is to widen the current perspective on basal ganglia connectomics providing a new challenging, comprehensive and integrated basal ganglia model. As previously mentioned, most of our knowledge on the basal ganglia is mainly based on invasive tract-tracing studies conducted on animals, whilst the available data on humans come from clinical evidences of patients with movement disorders and from pioneering neuroimaging studies.

**Figure 2:**
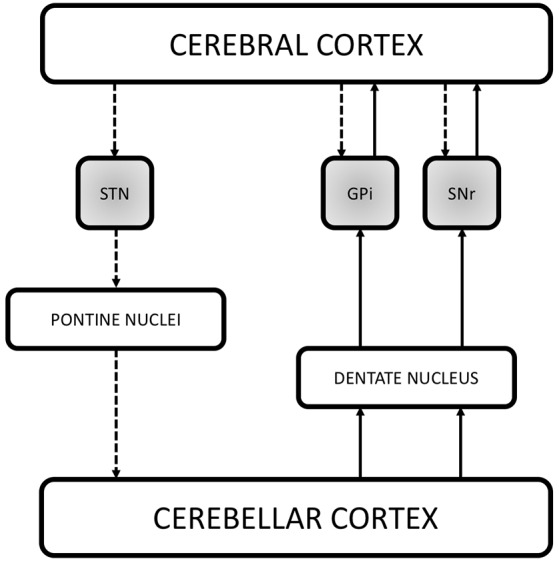
Schematic illustration of the recently demonstrated anatomical connections in the basal ganglia network. The figure reports the three direct systems running between the cerebral cortex and the basal ganglia (STN, GPi and SNr, shaded gray boxes), providing a fast route of connection by passing the striatum and the thalamus. Recent studies have also demonstrated that the basal ganglia communicate with the cerebellum. Retrograde transneuronal transport of rabies virus in monkeys revealed a disynaptic pathway from the STN passing through the pontine nuclei to the granule cells of the cerebellar cortex. Additional findings suggest the existence of reciprocal cerebellar output on the basal ganglia *via* the dentate nucleus. Indeed, it has been demonstrated both in animals and humans that the dentate nucleus is connected with the GPi and SNr thus directly influencing the output stations of the basal ganglia in the timing of actions as well as in action selection. The dashed lines represent the cerebral cortex output on the basal ganglia and the information flow from the basal ganglia to the cerebellum. The solid lines instead represent the cerebellar output on the output nuclei of the basal ganglia which in turn communicates with the cerebral cortex. STN, subthalamic nucleus; GPi, internal segment of the globus pallidus; SNr, pars reticulata of the substantia nigra.

The last 10 years have been characterized by the growing idea that, in addition to the direct, indirect and hyperdirect pathways, several other feedback and reverberating circuits can contribute to modulate basal ganglia output. Numerous studies have indeed pointed out that the basal ganglia directly integrate signals from widespread cortical areas and are part of an extensive network involving also the cerebellum (Figure 2).

### The Cortico-Pallidal Pathway

In a traditional textbook of anatomy, the French anatomist Testut remarked that “Ascending and descending cortico-caudatal, cortico-putaminal, and cortico-pallidal connections do exist. Cortico-caudatal and cortico-putaminal fibers are indicated together as cortico-striatal pathway: they are less than cortico-pallidal fibers. The cortico-pallidal fibers are prevalently but not exclusively cortico-fugal (efferent). These fibers (demonstrated both by anatomic dissection and by neuronography), originate from area 6” (Testut and Latarjet, 1971). Over the subsequent decades, the cortico-pallidal fibers almost disappeared from the literature. Early degeneration studies have described the possible existence of a direct cortico-pallidal projection in monkeys (Leichnetz and Astruc, 1977), leaving an open window to provide more conclusive evidences on the topic. By using BDA anterograde tract-tracing in rodents, Naito and Kita (1994) showed for the first time the existence of direct, topographically-organized connections between the medial and lateral precentral cortices and the GPe (Naito and Kita, 1994). Although it could be argued that these projections could represent passing fibers (that it is well known to be massively present in the GP), it is worthy to note that the BDA approach used in the study labeled with great precision fine fibers and boutons thus allowing to disentangle them from pallidal passing fibers. The existence of such fibers of passage could furthermore explain why retrograde tract-tracing techniques are not able to the show the presence of this cortico-pallidal pathway. Supporting evidences for the existence of such direct pattern of connectivity come from recent studies showing cholinergic and GABAergic neurons within the GPe that in turn send direct signals to the cerebral cortex (Chen et al., 2015; Saunders et al., 2015).

More recently, evidence supporting the likely existence of a direct cortico-pallidal pathway was provided by Milardi et al. (2015) using CSD-based tractography, thus being the first to characterize the cortico-pallidal connectivity patterns in the living human brain. More recently, Cacciola et al. (2017b) provided a quantitative connectomic analysis revealing that the pallidal network mainly involves the sensorimotor areas (i.e., precentral and postcentral gyri), the superior frontal and paracentral gyri, with less representative widespread connectivity patterns with other important cortical areas (Cacciola et al., 2017b). These findings have been further corroborated by other diffusion tractography studies (da Silva et al., 2017; Grewal et al., 2018; Middlebrooks et al., 2018; Cacciola et al., 2019).

Indirect evidences supporting a tight interplay between GP and frontal cortex in humans come also from PET studies in patients with focal lesions of the GP which have demonstrated reduced metabolism in frontal cortical areas as well as psychiatric symptoms reminiscent of patients with frontotemporal lobe damage. Taken together these findings strongly indicate a disrupted functional interaction between the GP and the frontal lobe (Laplane et al., 1989). In addition, by using a promising approach of simultaneous magnetoencephalography-local field potentials (MEG-LFP) recording in dystonic patients with deep brain stimulation (DBS) electrodes in the GPi, Neumann et al. (2015) demonstrated that the GPi is interconnected with several brain regions in spatial- and frequency-specific functional networks. In particular, MEG-LFP coherence analysis revealed oscillatory pallidal connectivity with the temporal cortex in the theta band (4–7 Hz), with the sensorimotor regions in the beta band (10–30 Hz) and with the cerebellum in the alpha band (6–13 Hz).

**Figure 3:**
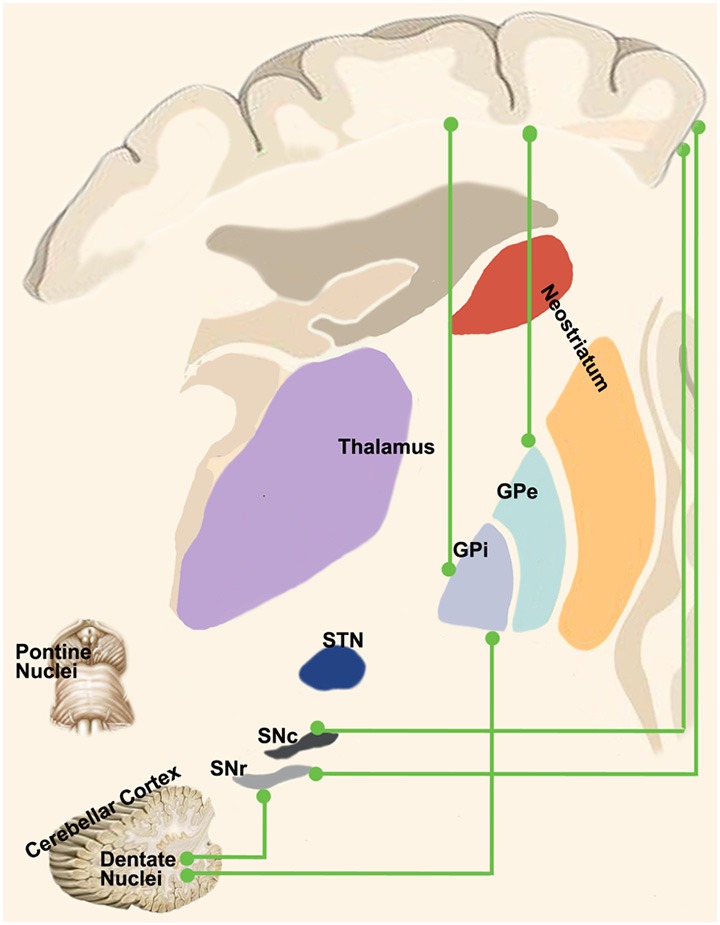
“Novel” cortico-basal-ganglia-cerebellar pathways. Highlight the newly identified connections between the cerebral cortex, GPi, GPe and SN as well as the complementary circuits between the dentate nucleus and such nuclei as described in recent tractographic studies in humans.

Therefore, the oscillatory drive of information flow between the motor-related areas and the GPi could be gathered either indirectly *via* the corticostriatal pathway or through a direct cortico-pallidal connection. The cortico-pallidal pathway could represent a possible anatomical substrate of the robust beta-band oscillatory activity in the cerebral-basal ganglia feedback loops involved in motor control (Cacciola et al., 2016b; Figure 3).

In addition, it has been reported in dystonic implanted patients, that single-pulse GPi-DBS may modulate motor cortical excitability at a relatively short latency suggesting the possibility of a direct cortical-GPi connection in humans (Cacciola et al., 2018; Ni et al., 2018b).

Recently, Cacciola et al. (2019) by using whole-brain tractography-based segmentation unveiled that the basal ganglia system is topographically organized in functionally segregated and integrated circuits within the GPi and GPe. In particular, the topographical organization of the cortico-pallidal pathway within the GP resulted in an antero-dorsal associative region and a posterior sensorimotor region, despite it was not possible to identify a well-defined limbic territory, thus suggesting that the cortico-pallidal fibers may provide only a relative contribution to the limbic territories in the GP. On the other hand, the most represented connectivity patterns to the GPi derived from sensorimotor regions suggesting a possible role of such pathway in sensorimotor integration. From a more practical point of view, this topographical segmentation of the GP applied to DBS, focused-ultrasound and radiosurgery interventions could improve patient’s outcome by minimizing side effects at the same time (Cacciola et al., 2019; Strafella, 2019).

Although the direct and indirect evidences on the possible existence of a monosynaptic pathway between the cerebral cortex and the GPi are continuously growing, its exact functional meaning is still not clear and speculative (Cacciola et al., 2018; Ni et al., 2018a, b).

### The Cortico-Nigral Pathway

Along with the GPi, the SNr is a key hub of the basal ganglia circuitry, involved in motor control (Friend and Kravitz, 2014), cognition (Simpson et al., 2010) and learning (Sesack and Grace, 2010), receiving both inhibitory and excitatory inputs from the striatum, GPe and STN, respectively (Kita and Kitai, 1987; Chevalier and Deniau, 1990; Smith et al., 1990). GABAergic neurons located in the SNr mainly target the peduncolopontine nucleus and the superior colliculi, thus suggesting SNr involvement in eyes, head and neck movements. In addition, SNr sends GABAergic inputs to the thalamic intralaminar nuclei that in turn send back projections to the striatum as well as to nuclei that send inputs to the cerebral cortex. In rodents, the ventromedial and paralaminar medial dorsal thalamic nuclei are the main target of GABAergic SNr inputs and in turn provide widespread projections to frontal cortical areas, including the equivalent eye field areas in primates. On the other hand, the principal targets of the SNr are the ventral anterior and paralaminar medial dorsal nuclei which instead project to more discrete organized frontal areas (Bentivoglio et al., 1979; Hoover and Strick, 1999).

By contrast, both in rodents and in primates, SNc provides extensive dopaminergic innervation to dorsal and ventral striatum (Beckstead et al., 1979; Haber, 2014). From striatum in turn, originates a set of reciprocating GABA-ergic connections to SNc (Szabó, 1979; Haber et al., 2000). In addition to these projections, SNc receives excitatory glutamatergic afferents from the STN, and GABA-ergic projections from GPi and SNr (Smith and Kieval, 2000; Watabe-Uchida et al., 2012).

Therefore, both the SNc and SNr receive disynaptic inhibitory and excitatory inputs from the cerebral cortex *via* the neostriatum and STN respectively. In addition, several anatomical studies have indicated a direct connection between the cortex and the SN (Figure 3). Although the majority of these studies have clearly shown the existence of a direct cortico-SN pathway, the topographical arrangement, the extent of the cortical regions involved in the projection and the morphological characteristics of the fibers and boutons were not well clarified until the mid-nineties. In an anterograde tracing study with BDA in rats, Naito and Kita (1994) addressed this issue by showing that the SNc received orderly arranged, but sparse connections from the entire prefrontal cortex; the density of boutons in SNc was much less than the ones of the striatum. More recently, Frankle et al. (2006) injected anterograde tracers into the orbital (OFC), cingulate and dorsolateral prefrontal (dlPFC) cortices, demonstrating direct connections from OFC and dlPFC to SN in the macaque monkey.

In human, the SN is involved in an extensive sub-cortical network (Düzel et al., 2009; Menke et al., 2010; Chowdhury et al., 2013), despite less is known about the possible existence of a human homologous of the direct cortico-nigral connections demonstrated in animals. In this regard, by using dMRI and tractography, Cacciola et al. (2016a) have recently reconstructed a white matter pathway linking the superior frontal, inferior frontal, precentral, postcentral gyri and the paracentral lobule with the SN bypassing the caudate nucleus, the putamen, the GP and the STN in the human brain (Cacciola et al., 2016a). In addition, in line with previous findings, the same authors demonstrated that the SN is extensively connected with many sensorimotor and associative cortical areas as well as with subcortical structures, including the cerebellum (Cacciola et al., 2017b).

In conclusion, the basal ganglia connectome seems to be more complex than expected; non-canonical pathways such as the cortico-pallidal and cortico-nigral pathways may have a role in basal ganglia physiology and pathophysiology of basal ganglia disorders. However, their functions remain speculative and need more investigation to be completely understood.

## The Cerebellum and Basal Ganglia Interplay

Along with the fundamental role in motor control, the cerebellum and basal ganglia are involved in several aspects of behavior, from cognition to emotion (Middleton and Strick, 1994; Schmahmann and Caplan, 2006). The involvement of the cerebellum in so many functions could be explained by taking into account that it works in strict connection with the cerebral cortex and the basal ganglia, which in turn play both a pivotal role in a variety of motor and non-motor functions.

According to the traditional view, the cerebellum and basal ganglia interact at the level of the cerebral cortex. However, the last decades have been characterized by increasing evidences showing a direct cerebello-basal ganglia interplay forming an integrated building block involved in several complex tasks.

Anterograde and retrograde studies demonstrated that neurons of the central lateral nucleus of thalamus, which projects both to motor cortex and to laterodorsal part of the striatum, receive inputs from the lateral cerebellar nucleus (Ichinohe et al., 2000). These findings were extended to non-human primates in a study conducted on macaques by means of retrograde transneuronal transport of rabies virus (Hoshi et al., 2005), showing a pathway linking primarily the dentate nucleus (but also the interpositus and fastigial nuclei) to the contralateral striatum through ventral anterior (VA), ventral lateral (VL) and intralaminar nuclei (CM/Pf) and finally reaching the external part of the globus pallidus (Figure 3). Labeled neurons in the dentate nucleus belonged both to its motor and non-motor domains (Dum et al., 2002), suggesting that the interplay of these subcortical structures is crucial for motor, cognitive and emotional processing.

A few years later, Bostan et al. (2010) employed the same experimental setting to investigate the presence of a pathway projecting from basal ganglia to cerebellum. The retrograde transport revealed that first-order neurons were located in the pedunculopontine nucleus while second-order neurons were found to be topographically organized in the STN (Figure 3). These fascinating studies provided new insights on the roles of basal ganglia and cerebellum showing that their interplay may be more complex than expected. Virus tracing is not the only technique which has been employed to study connectivity between these two subcortical structures. Converging evidences coming from electrophysiological experiments and human neuroimaging studies will be discussed below.

## Electrophysiological Insight Into Cerebellar-Basal Ganglia Interactions

Electrophysiological investigations, conducted on anesthetized cats to assess the latency of basal ganglia-cerebellum activation, failed in finding strong evidences of a rapid-gated cerebellum-basal ganglia communication. The long latencies (50–350 ms) found made the hypothesis of rapidly funneling stimuli from cerebellum to basal ganglia neglectable (Ratchetson and Li, 1969). This assumption has been recently challenged by Chen et al. (2014) in a optogenetic study on freely moving rats, which revealed short-latency activation (10 ms) of basal ganglia after optogenetic stimulation of dentate nucleus, thus accounting for a rapid communication between cerebellum and basal ganglia leading to fine coordination of their respective outputs. Moreover, when the electrical stimulation of dentate nucleus is delivered simultaneously to high frequency stimulation of cerebral cortex, the overall result is a direction change of synaptic plasticity, reverting long term depression (LTD) in long term potentiation (LTP; Chen et al., 2014). These findings do provide new insight on the role of basal ganglia-cerebellum communication in learning phenomena. The synergic role of cerebellum and basal ganglia in learning processes is not new considering the pioneer studies of the early 2000 showing that the cerebral cortex, cerebellum and basal ganglia are involved in specific learning paradigms: unsupervised, error-based (supervised) and reward-based learning (Doya, 1999, 2000). The recent anatomical findings of the two- and tri-synaptic pathways linking the cerebellum and basal ganglia, together with the evidence of a short latency communication, led Caligiore et al. (2017) to consider their computational role and to update the previous model of the cortico-basal ganglia-cerebellum loops. The possible computational role of the dento-thalamo-striatal pathway is to convey the predicted outcome of a candidate action, processed in the cerebellum to the striatum where the outcome itself is evaluated (*forward model*). On the other hand, the computational role of the subthalamic-ponto-cerebellar pathway is not clear at all; nevertheless, considering the involvement of the subthalamic nucleus in the indirect pathway and aversive learning phenomena, it is tempting to speculate that it would prevent the new *forward models* to be conveyed to the striatum (Caligiore et al., 2017).

## Possible Direct Cerebellar-Basal Ganglia Connections

In addition to the dento-thalamo-striatal and subthalamo-ponto-cerebellar pathways, Milardi et al. (2016a) reconstructed a white matter pathway linking the dentate nucleus both to the GPi and to the SN *via* the superior cerebellar peduncles and bypassing the red nucleus, thalamus and striatum (Milardi et al., 2016a; Figure 3).

Although its physiological meaning is still unknown, the dento-nigral pathway, reconstructed in human by means of dMRI and tractography, could represent the phylogenetical equivalent of the pathway observed *via* tract-tracing in cats and rats (Snider et al., 1976) allowing a fine-tuning of a fast cerebellar influence of one of the output nuclei of the basal ganglia system. In addition, release of dopamine in caudate nucleus and incremented dopamine production in substantia nigra were found after unilateral stimulation of the dentate nucleus in cats (Nieoullon et al., 1978) suggesting that direct connections from deep cerebellar nuclei could exert a modulatory role on dopaminergic tone in the basal ganglia.

Recent evidences of a direct route connecting dentate nucleus to globus pallidus, on human side, comes from a MEG-LFP study (Neumann et al., 2015), showing a functional oscillatory connectivity in the alpha band (7–13 Hz) between the cerebellum and globus pallidus in dystonic patients with an electrode implanted in the GPi. In addition, it was also found a negative correlation between the alpha band of coherence and symptoms severity as measured by Toronto Western Spasmodic Torticollis Rating Scale suggesting a compensatory role of the cerebellum in dystonic patients.

**Figure 4:**
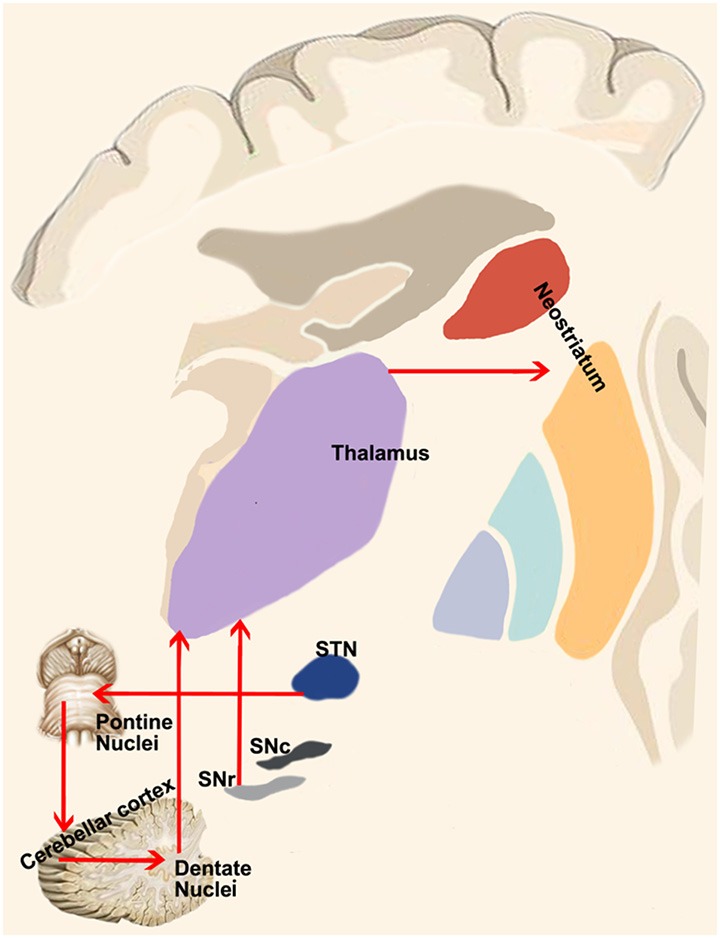
Cerebellum-basal ganglia interplay. This panel shows the connections between the cerebellum and basal ganglia as revealed by retrograde tracing studies in monkeys. Red lines indicate the output of the cerebellum on the basal ganglia *via* the dentate-thalamo-striatal pathway as well as the control of basal ganglia on the cerebellum *via* the STN-ponto-cerebellar cortex pathway.

These direct connections between the dentate nucleus and GPi and SNr are very intriguing considering the presence of a direct cortico-pallidal and cortico-SN pathways bypassing the striatum in humans (Milardi et al., 2015; Cacciola et al., 2016a) and in monkeys (Leichnetz and Astruc, 1977; Frankle et al., 2006). Hence, it is tempting to speculate on the existence of 3 direct systems running between the cortex, the basal ganglia (STN, GPi and SNr) and the cerebellum, providing a fast route of connection bypassing the striatum and the thalamus (Figure 4). These considerations are not necessarily in conflict with the consensus position of Caligiore et al. (2017) if we postulate the appearance, in the evolutionary scale in humans, of a new phylogenetic fast system connecting cerebellum and basal ganglia which may complement the disynaptic or trisynaptic projections from the dentate nucleus, passing through the thalamus and reaching the putamen or the GPe. This new fast system would be necessary to support the manual dexterity which is an exquisite feature of human specimens.

## Functional Significance of Cerebellar-Basal Ganglia Interaction in Movement Disorders

The basal ganglia and the cerebellum have been often conceived separately as structures involved in different neurological syndromes. However, evidences concerning the co-operation of cerebellum and basal ganglia in movement disorders are currently growing. Thus, the above-described scenario could open an entirely new perspective into the pathophysiology of basal ganglia and cerebellum disorders (Coenen et al., 2011; Husárová et al., 2014).

Different aspects of movement disorders could be gathered by cerebellum-basal ganglia interface. Cerebellum and basal ganglia have been involved in time computation: the former should be accounted for millisecond-range intervals whilst the latter would work mainly on the second-ranges (Ivry, 1996; Buhusi and Meck, 2005; Wiener et al., 2010). Functional MRI (fMRI) studies revealed hypoactivation of basal ganglia and cerebellar cortex during early stages of Parkinson’s Disease (PD) compared to healthy controls, during an interception task. A direct causal modeling analysis revealed differential modulation of effective connectivity strength between basal ganglia and cerebellum in performing a motor timing task (Husárová et al., 2013, 2014). This would suggest an involvement of cerebello-basal ganglia circuits in motor and perceptual timing alterations, that are typical of PD.

Although the involvement of basal ganglia in the pathophysiology of dystonia is indisputable, the mechanisms producing dystonia are incompletely understood, with recent evidence pointing to the involvement of a variety of brain areas including the cerebellum (Quartarone and Hallett, 2013; Jinnah et al., 2017). As it is possible that the etiological heterogeneity of dystonias reflects the relative importance of different nodes in this extended motor network, one major challenge is determining first, the role and contribution of the different brain regions in the various forms of dystonia with a comprehensive model; second, if there is a final common pathway for all dystonias (Quartarone and Ruge, 2018).

Anomalies in the cerebellum and basal ganglia have been widely investigated in both animal and human studies of dystonia (Filip et al., 2013; Tewari et al., 2017). Different cases of secondary dystonia emerging from cerebellar lesions are described in humans (Alarcón et al., 2001; LeDoux and Brand, 2003; Shen et al., 2016). In a murine model of cerebellar-induced dystonia, a cerebellar outflow interruption has been causally linked to burst firing activity in basal ganglia, which is a prominent feature of dystonia (Chen et al., 2014).

Moreover, also primary dystonia, such as cervical dystonia has also been conceptualized as deriving from alterations in neural integration for head and eye movements, involving cerebellum and basal ganglia in association with oculomotor structures (Shaikh et al., 2016). In line with this hypothesis, in a fMRI study during a visuospatial task, Filip et al. (2017) observed cerebellum-basal ganglia hypoconnectivity in patients with cervical dystonia.

The pathway linking the STN to cerebellum could be involved in motor symptoms of PD. In particular, STN pathological activity, characterized by burst activity and higher firing rates, may in turn be responsible for hyperactivity of cerebellar cortex leading to alterations in the cerebello-thalamo-cortical circuits (Bostan et al., 2010; Bostan and Strick, 2018). It can be therefore hypothesized that DBS of the STN could exert its positive effects on motor learning by stimulating the pathway linking the STN to the cerebellum. Supporting this hypothesis, a recent fMRI study on 20 PD patients implanted with DBS of the STN found that functional connectivity between active contact and contralateral cerebellum is strongly predictive of improvement in motor learning (de Almeida Marcelino et al., 2019). It would be tempting to speculate that suppression of STN aberrant activity, promoted by DBS, could lead to improved cerebellar function and, by consequence, to improvement in motor learning.

## Conclusions

In conclusion, further experimental and challenging studies should be fostered to characterize the full extent of the interplay between the cerebral cortex, the basal ganglia and the cerebellum. However, several evidences have already suggested that the system is more intricated than initially assumed. In the present review, we discussed the invasive and non-invasive techniques to investigate the anatomy and the extrinsic and intrinsic connections of the basal ganglia network. We illustrated the neuroanatomical findings obtained in non-human species that have inspired a paradigmatic shift in this scenario, providing evidences that the cortico-basal ganglia circuits constitute a complex system. Finally, we provide further support coming from neuroimaging studies that these pathways may exist in humans and may exert a meaningful role in basal ganglia disorders. Taken together, these observations suggest that the cerebral cortex, the basal ganglia and the cerebellum form an integrated and segregated network acting on multiple motor and non-motor functions. Although such complex interplay has not yet been explored in detail, we hope it will be a focus of new-generation optogenetic, physiologic, behavioral and neuroimaging studies.

The proposed scenario, with the presence of parallel direct and indirect projections running between the cortex, basal ganglia and cerebellum, complements new ideas that view movement disorders as disorders of a complex motor network rather than a limited disruption of individual nuclei in the basal ganglia.

## Author Contributions

DM: writing of the first draft of the manuscript, conception, organization, and execution of the research project, data analysis and interpretation, literature research, and critically revised the manuscript. AQ: writing of the first draft of the manuscript, conception, organization, and execution of the research project, data analysis and interpretation, literature research, critically revised the manuscript, and Guarantor and supervisor of the research project. GA: conception, organization, and execution of the research project, data interpretation, critically revised the manuscript, Guarantor and supervisor of the research project. AC: conception, organization, and execution of the research project, data analysis and interpretation, writing of the first draft and revision of the final version of the manuscript, and Guarantor and supervisor of the research project. AB, SB, GB, PB and GP: data analysis and interpretation, writing of the first draft and literature research. GC: data analysis and interpretation, revised the manuscript and literature research. GR and DB: data analysis and interpretation and literature research.

## Conflict of Interest

The authors declare that the research was conducted in the absence of any commercial or financial relationships that could be construed as a potential conflict of interest.
